# Structural basis for assembly of non-canonical small subunits into type I-C Cascade

**DOI:** 10.1038/s41467-020-19785-8

**Published:** 2020-11-23

**Authors:** Roisin E. O’Brien, Inês C. Santos, Daniel Wrapp, Jack P. K. Bravo, Evan A. Schwartz, Jennifer S. Brodbelt, David W. Taylor

**Affiliations:** 1 Institute for Cell and Molecular Biology, University of Texas at Austin, Austin, TX 78712 USA; 2 Department of Chemistry, University of Texas at Austin, Austin, TX 78712 USA; 3 Department of Molecular Biosciences, University of Texas at Austin, Austin, TX 78712 USA; 4 Center for Systems and Synthetic Biology, University of Texas at Austin, Austin, TX 78712 USA; 5 Livestrong Cancer Institutes, Dell Medical School, Austin, TX 78712 USA

**Keywords:** Enzyme mechanisms, RNA, Cryoelectron microscopy, CRISPR-Cas systems

## Abstract

Bacteria and archaea employ CRISPR (clustered, regularly, interspaced, short palindromic repeats)-Cas (CRISPR-associated) systems as a type of adaptive immunity to target and degrade foreign nucleic acids. While a myriad of CRISPR-Cas systems have been identified to date, type I-C is one of the most commonly found subtypes in nature. Interestingly, the type I-C system employs a minimal Cascade effector complex, which encodes only three unique subunits in its operon. Here, we present a 3.1 Å resolution cryo-EM structure of the *Desulfovibrio vulgaris* type I-C Cascade, revealing the molecular mechanisms that underlie RNA-directed complex assembly. We demonstrate how this minimal Cascade utilizes previously overlooked, non-canonical small subunits to stabilize R-loop formation. Furthermore, we describe putative PAM and Cas3 binding sites. These findings provide the structural basis for harnessing the type I-C Cascade as a genome-engineering tool.

## Abstract (web summary)

Type I-C Cascade (the CRISPR-associated complex for antiviral defense) is a minimal system, comprising only three unique Cas proteins. Cryo-EM structure of the *Desulfovibrio vulgaris* type I-C Cascade reveals the molecular mechanisms that underlie RNA-directed complex assembly.

## Introduction

CRISPR-RNA (clustered, regularly, interspaced, short palindromic repeats-RNA) along with Cas proteins assemble into RNA-guided adaptive immune complexes in prokaryotes^1^. These CRISPR–Cas systems defend bacteria and archaea against the invasion of foreign genetic elements^2^. CRISPR–Cas systems can be divided into two major classes based on their targeting complexes: multi-subunit effector (Class I) or a single protein effector (Class II)^3^. The type I-C subtype is one of the most prevalent systems found in bacteria^4^. However, relatively little information exists about its effector complex.

**Fig. 1:**
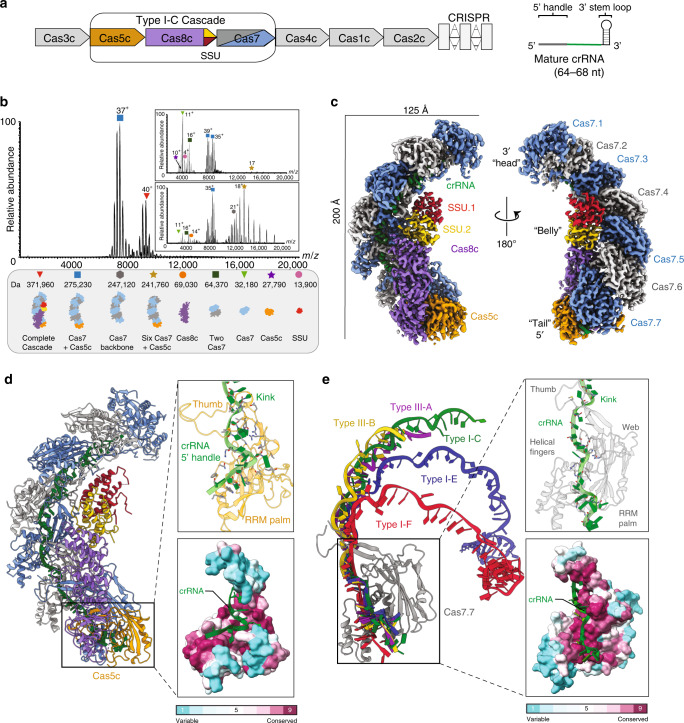
Architecture of type I-C Cascade. **a** The *D. vulgaris* type I-C operon (left) and mature crRNA (right). **b** ESI mass spectrum of the type I-C Cascade confirms the presence of a non-canonical SSU. Gentle disassembly of the complexes prior to mass analysis using two different desolvation voltages (−120 and −300 V) allows the composition of the individual subunits and the architecture of the complexes to be determined (inset top and bottom). All complexes/subcomplexes detected and associated experimental masses are annotated. **c** Cryo-EM structure of the type I-C Cascade at 3.1-Å resolution. The structure is segmented and colored as follows: Cas7, blue and gray; Cas8c, purple; Cas5, orange; Cas11c, gold and red; crRNA, green. **d** The resulting atomic model colored as in **c**. Cas5c (orange) resembles a right-handed fist with an RRM palm and thumb domains. Residues (gray) interacting with the 5′-handle (green) (inset top) are highly conserved (inset bottom). **e** Alignment of type III-B (yellow), type I-E (blue), type I-F (red), type III-A (purple) Cas7 tail to type I-C Cas7.7 demonstrates variability in crRNA backbone orientation. Residues (dark gray) interacting with the crRNA (green) (inset top) are highly conserved (inset bottom).

Interestingly, type I-C Cascade only contains three unique Cas proteins in its operon: Cas5c, Cas7, and Cas8c^3^ (Fig. 1a). The type I-C Cascade uses Cas5c for processing the crRNA instead of a separate Cas6 (refs. ^3,5,6^) and does not include a small subunit (SSU) within its operon^3^, making this a minimal Cascade (Fig. 1a). Previous studies hypothesized that the large subunit, Cas8c, was a fusion of the larger and smaller subunits found in the type I-E Cascade^6^. However, a recent report revealed that the *Desulfovibrio vulgaris* Cas8c large subunit includes an internal ribosome-binding site at the C terminus, which encodes a separate SSU^7^. This non-canonical SSU was shown to be equivalent to the Cas11 SSU found in type I-E and appeared widespread within the I-B, I-C, and I-D subtypes^7^. Here, we demonstrate that this non-canonical subunit is an integral component within the complex and is primed for stabilizing the non-target strand during R-loop formation.

## Results

### Stoichometry, assembly, and cryo-electron microscopy (cryo-EM) structure of type I-C Cascade complex

We purified the *D. vulgaris* type I-C Cascade from *Escherichia coli*, which revealed the presence of an additional 14 kDa protein, corresponding to the recently identified SSU (Supplementary Fig. 1). We then analyzed the complex using native mass spectrometry (MS)^8–11^, which exhibited the presence of two dominant species with masses of 275 and 371 kDa, respectively (Fig. 1b). The larger species (371 kDa) corresponds to a fully intact type I-C Cascade with a stoichiometry of Cas7_7_Cas8c_1_Cas5c_1_SSU_2_/crRNA_1_. The smaller species (275 kDa) is consistent with the Cascade lacking Cas5c and Cas8c or lacking the two SSUs and Cas8c. Since previous isothermal titration calorimetry experiments^6^ have demonstrated that Cas5c has a higher affinity for the crRNA than Cas8c; the 275 kDa subcomplex most likely represents Cascade after dissociation of the SSUs and Cas8c due to weakening of hydrophobic interactions within the gas phase^12^. Application of gentle collisional activation via in-source trapping (IST) was used to disassemble the complexes prior to mass analysis, thus allowing inspection of the composition of the individual subunits and the architecture of subcomplexes (Fig. 1b, insets). The theoretical and experimental masses obtained from native mass spectra with IST are provided in Supplementary Table 1.

To understand the molecular basis for small-subunit incorporation, we determined a 3.1 Å resolution cryo-EM reconstruction of the type I-C Cascade complex (Fig. 1c, Supplementary Figs. 2–4, and Supplementary Table 2), suitable for de novo model building (except for the flexible N terminus of Cas8c) (Supplementary Fig. 5). The overall architecture of the complex resembles a caterpillar. Seven Cas7 subunits form a right-handed helical filament around the crRNA and Cas5c sits at the base of the complex (Fig. 1d). Cas5c and Cas7.7 clamp around the crRNA 5′-handle (nucleotides U1–G12), forcing it into a hooked conformation (Fig. 1d, inset). Cas5c residues “pinch” the phosphate groups within the crRNA backbone on either side of the U5 nucleobase, inducing a sharp (33°) kink. Nucleotides on either side of this kink are captured by a network of Cas5c π–π stacking interactions, while Cas7.7 makes non-specific contacts with the phosphate backbone (Supplementary Fig. 6). These highly conserved interactions (Fig. 1d, inset) suggest that the 5′ end of the crRNA handle is critical for type I-C Cascade assembly.

Seven Cas7 subunits span the length of the crRNA and are capped by the 3′ end (Fig. 1c). While type I-E and type I-F Cascades incorporate a Cas6 subunit, an additional Cas7 subunit forms the head of the type I-C Cascade^13,14^ (Fig. 1c). Interestingly, when the bottom Cas7 subunits from type I-F, I-E, IIII-A, and III-B are all aligned to the type I-C Cas7.1, the type I-C crRNA backbone more closely resembles that of type III-A and -B complexes (root-mean-square deviation (RMSD) 7.8 Å), rather than the type I-E (RMSD 10.6 Å) or type I-F (RMSD 19.1 Å) Cascades (Fig. 1e)^8–11^. The type III-A, type III-B, and type I-C crRNA lack a 3′ stem–loop, which correlates with a more linear geometry of the crRNA backbone^15,16^ (Fig. 1e). Despite these differences, type I-C Cas7 maintains a highly conserved region of positive residues to form non-specific interactions with the phosphate backbone of the crRNA. (Fig. 1e, inset, and Supplementary Fig. 7).

**Fig. 2:**
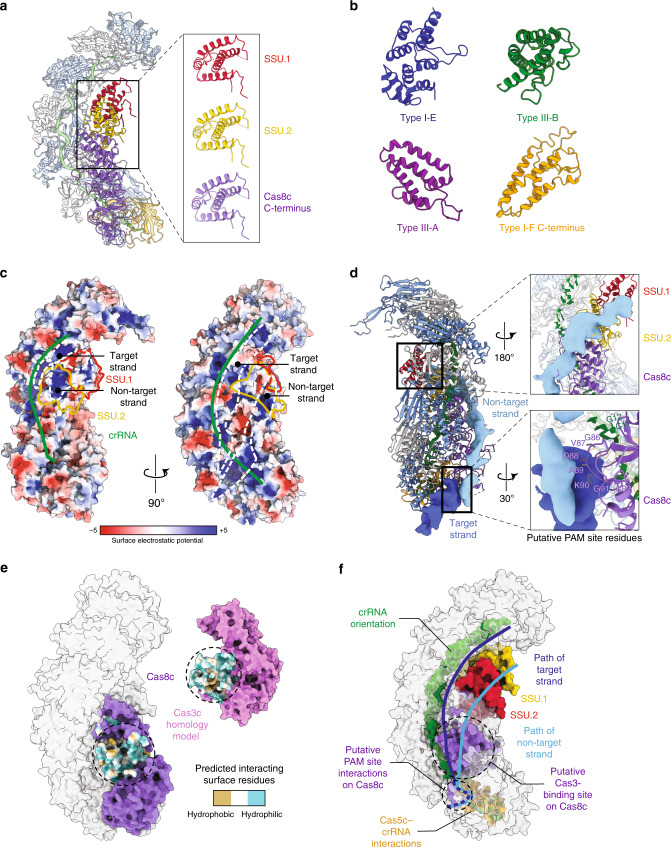
Non-canonical small subunits (SSU) support R-loop formation. **a** Cas11 SSU are identical to the C terminus of Cas8c. **b** Canonical SSU found in type I-E, type III-A, type III-B, and the C-terminal domain of the type I-F large subunit aligned to the type I-C SSU. These SSUs are all helical bundles. **c** A channel of positively charged surface residues show Cas11c SSU likely accommodate the non-target strand during R-loop formation. **d** Putative Cas8c (purple) PAM site interacting residues (gray) determined by overlaying the dsDNA density (blue) from previous type I-C cryo-EM maps. **e** Cas8c (purple) and a homology model Cas3c (pink) show complementary surfaces for Cas3 docking. **f** Model of type I-C Cascade function.

The belly of the complex contains the large subunit, Cas8c, and two copies of the SSU, which nucleate and are derived from the C-terminal domain of Cas8c (residues 489–612) (Fig. 1c). These SSUs are structurally identical to the C-terminal domain of Cas8c (RMSD of 0.59 and 0.67 Å for SSU.1c and SSU.2c to Cas8c C terminus, respectively) (Fig. 2a) and adopt a helical bundle topology typical of other SSUs^8–11^ (Fig. 2b). In the type I-E system, the Cse2 SSUs are responsible for supporting the non-target strand during R-loop formation (Supplementary Fig. 8). Remarkably, the electrostatic surface potential of the type I-C Cascade (Fig. 2c) reveals a contiguous channel of positively charged residues that runs along the length of this minor filament from the large subunit (Fig. 2c). We then compared our model with a previous lower-resolution reconstruction of type I-C Cascade^6^ (Fig. 2d). As anticipated, additional density corresponding to the non-target strand follows the positively charged path across the surface of the SSU (Fig. 2d, inset), indicating that these non-canonical SSUs may accommodate the non-target strand during DNA targeting.

### Structural insights into PAM recognition and Cas3c recruitment

In the type I-E Cascade, the large subunit Cse1 is responsible for identifying the PAM (protospacer adjacent motif) site on the non-target strand of the dsDNA target^17–19^. Notably, the overlay of the target DNA density shows Cas8c is in a position to interact with the PAM sequence in the duplex. A glycine loop and adjacent positively charged residues create a putative PAM binding site (Fig. 2d, inset) located near position 1- and 0-nt (C11 and G12), which are required for target recognition. Following PAM recognition, a trans-acting nuclease-helicase Cas3 subunit is recruited for target degradation in most type I systems, and interacts exclusively with the large subunit^20–22^. To understand Cas3c recruitment, we generated a homology model of Cas3c and predicted its Cas8c-interacting surfaces using MorphProt^23^, revealing regions of complementary charges and hydrophobicity located on the surface of Cas8c and Cas3c (Fig. 2e). This binding site positions Cas3c to favorably interact with the non-target strand during R-loop formation (Fig. 2f) and is consistent with previously reported Cas3-bound Cascade structures^21^.

## Discussion

Our structural work provides the first molecular insights into the sequence-specificity of Cas5c–crRNA interactions and non-specific Cas7–crRNA interactions that are critical for type I-C Cascade assembly. The Cas5c-Cas7.7 clamp around the crRNA nucleates Cascade complex assembly, which is likely followed by cooperative assembly of the Cas7 backbone. This culminates in the addition of the Cas8c–Cas11.1c–Cas11.2c “belly” architecture. This hierarchical assembly is supported by our native MS data, which demonstrate that Cas5c–Cas7–crRNA form a stable complex in the absence of Cas8c and Cas11c (Fig. 1b). We reveal how the incorporation of a previously overlooked SSU may stabilize the non-target strand during R-loop formation. Furthermore, we identify distinct, exposed surfaces on Cas8c that creates a central hub for DNA duplex separation, PAM recognition, Cas3c recruitment, and ultimately dsDNA degradation by the minimal type I-C Cascade (Fig. 2f). Taken together, our model provides functional insights into one of the most prevalent CRISPR–Cas systems in bacteria which may serve as a blueprint for developing a minimal Cascade for genome editing^24,25^.

## Methods

### Protein purification

The *D. vulgaris* type I-C Cascade (addgene plasmid #81185) and its crRNA (addgene plasmid #81186) were co-expressed in NiCo21(DE3) *E. coli* cells. Cells were grown at 37 °C to an OD_600_ of 0.6–0.8 and induced by the addition of 0.5 mM isopropyl-β-d-thiogalactopyranoside. After overnight growth at 18 °C, the cells were harvested and lysed by sonication in a buffer containing 50 mM HEPES–NaOH (pH 7.5), 500 mM KCl, 10% (v/v) glycerol, 1 mM tris(2-carboxyethyl)phosphine (TCEP), 0.01% Triton X-100, 0.5 mM PMSF, and complete mini protease inhibitor tablets. The lysate was centrifuged at 27,000 × *g* and incubated with Ni-NTA affinity resin overnight. The protein-bound resin was centrifuged and washed with buffer containing 50 mM HEPES–NaOH (pH 7.5), 500 mM KCl, 20 mM imidazole, 10% (v/v) glycerol, and 1 mM TCEP. Protein was eluted with 50 mM HEPES–NaOH (pH 7.5), 500 mM KCl, 300 mM imidazole, 10% (v/v) glycerol, and 1 mM TCEP. Approximately 1 mg of TEV protease was added per 25 mg of protein and the protein-TEV mixture was dialyzed at 4 °C overnight against size-exclusion buffer. The protein was then concentrated to approximately 10 mg/mL and run over a Superdex 200 Increase 10/300 GL size-exclusion column in a buffer containing 50 mM HEPES–NaOH (pH 7.5), 500 mM KCl, 5% (v/v) glycerol, and 1 mM TCEP. The proteins were analyzed for purity by 10–20% SDS-Page (Fig. S1) and then dialyzed overnight into the storage buffer containing 20 mM HEPES–NaOH (pH 7.5), 100 mM KCl, 5% (v/v) glycerol, and 1 mM TCEP. All proteins were finally concentrated, flash frozen in liquid nitrogen, and stored at −80 °C. Source data is provided in the source data file.

### Mass spectrometry

Prior to mass spectrometric analysis, the CRISPR complex solution buffer was exchanged to 100 mM ammonium acetate using Micro Biospin P-6 gel columns (Bio-Rad Laboratories Inc., Hercules, CA). MS measurements were performed in positive mode using a Thermo Scientific Q Exactive Plus UHMR instrument (Bremen, Germany). Samples were loaded into gold/palladium-coated borosilicate capillaries fabricated in-house. An electrospray voltage of 1.0 kV was applied. The concentration of the CRISPR complex in solution was estimated as ~6 μM. Trapping gas pressure was set to 10 (~1.0 × 10^−9^ mbar) for high mass analysis and to 1–3 (~1.0 × 10^−10^−2.5 × 10^−10^ mbar) for low mass analysis. For the detection of the subunits, the in-source-trapping voltage (ranging from −100 to −300 V) was optimized for the release and transmission of the individual proteins as well as subcomplexes. In order to trap the macromolecular complexes, lower RF amplitudes of the bent flatapole and injection flatapole (range of 300 V instead of 900 V) and IST voltages (−120 and −300 V) were used. MS1 and in-source trapping mass spectra were decharged and deisotoped using Xtract with a signal-to-noise ratio of 2, fit factor of 44%, and remainder of 25%. Additionally, raw spectra were deconvoluted using UniDec^26^.

### Cryo-EM sample preparation and data collection

Purified type I-C Cascade was diluted to a concentration of 0.3 mg/mL in a buffer containing 20 mM HEPES–NaOH (pH 7.5), 100 mM KCl, and 1 mM TCEP. The CF-2/2 grids were first glow discharged for 60 s and then a layer graphene oxide was added^27,28^. Three microliters of protein were deposited on the grid and excess protein was blotted away after a 0.5 s incubation time for 4 s using filter paper at 4 °C in 100% humidity. The grid was then plunge frozen into liquid ethane using a Vitrobot Mark IV (Thermo Fisher). Frozen-hydrated samples of type I-C Cascade were directly visualized using a FEI Titan Krios microscope equipped with a Gatan K3 direct electron detector. Using the automated data-collection software LEGINON^29^, we acquired ~5400 movies at a magnification of ×22,500, corresponding to a calibrated pixel size of 1.047 Å/pixel. A full description of the cryo-EM data collection parameters can be found in Table S2.

### Cryo-EM data processing

Motion correction, CTF (contrast transfer function) estimation, and non-templated particle picking were performed in Warp^30^. Extracted particles were imported into CryoSPARC^31^ for 2D classification, 3D classification, and non-uniform 3D refinement. The final reconstruction was sharpened in CryoSPARC and subjected to density modification in PHENIX^32,33^. A final structure of type I-C Cascade at 3.13-Å resolution was determined using the 0.143 gold standard Fourier shell correlation—calculated from two independent half-sets—criterion. The model was built de novo in Coot^34^, and refined in PHENIX, ISOLDE^35^, and NAMDINATOR^36^. The full cryo-EM data processing workflow is described in Fig. S2, and the model refinement statistics can be found in Table S2 and Fig. S3.

### Reporting summary

Further information on research design is available in the Nature Research Reporting Summary linked to this article.

## Supplementary information

Supplementary Information

Reporting Summary

## Data Availability

The data that support the findings of this study are available from the corresponding author upon request. The cryo-EM structure of the type I-C minimal Cascade have been deposited into the Electron Microscopy Data Bank with accession number EMD-22876. The associated atomic models have been deposited into the Protein Data Bank with PDB code 7KHA. Source data are provided with this paper.

## Source data

Source Data
