# Supplementary material for: Structural basis for assembly of non-canonical small subunits into type I-C Cascade
Source: Nat Commun. 2020 Nov 23;11:5931. doi: 10.1038/s41467-020-19785-8 (PMC7684278; doi:10.1038/s41467-020-19785-8)
Supplement: Supplementary file 1 — Supplementary Information [file 41467_2020_19785_MOESM1_ESM.pdf]

Supplementary Information

Structural basis for assembly of non-canonical small subunits into type I-C Cascade

Roisin E. O'Brien, Inês C. Santos, Daniel Wrapp, Jack P.K. Bravo, Evan A. Schwartz, Jennifer S. Brodbelt, and David W. Taylor

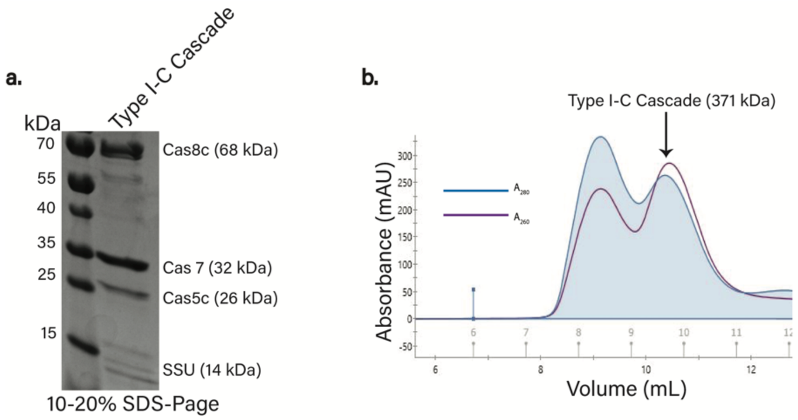

Supplementary Figure 1. *D. vulgaris* Type I-C Cascade expression and purification. a, SDS-Page analysis of the type I-C Cascade. This experiment was repeated once. Source data are provided in the source data file. **b,** Size-exclusion chromatogram of the affinity-purified type I-C Cascade. Data from the Superdex 200 Increase 10/30 GL columns is shown in blue (nucleic acid) and purple (protein). The elution of the 371 kDa minimal Cascade complex is shown by an arrow.

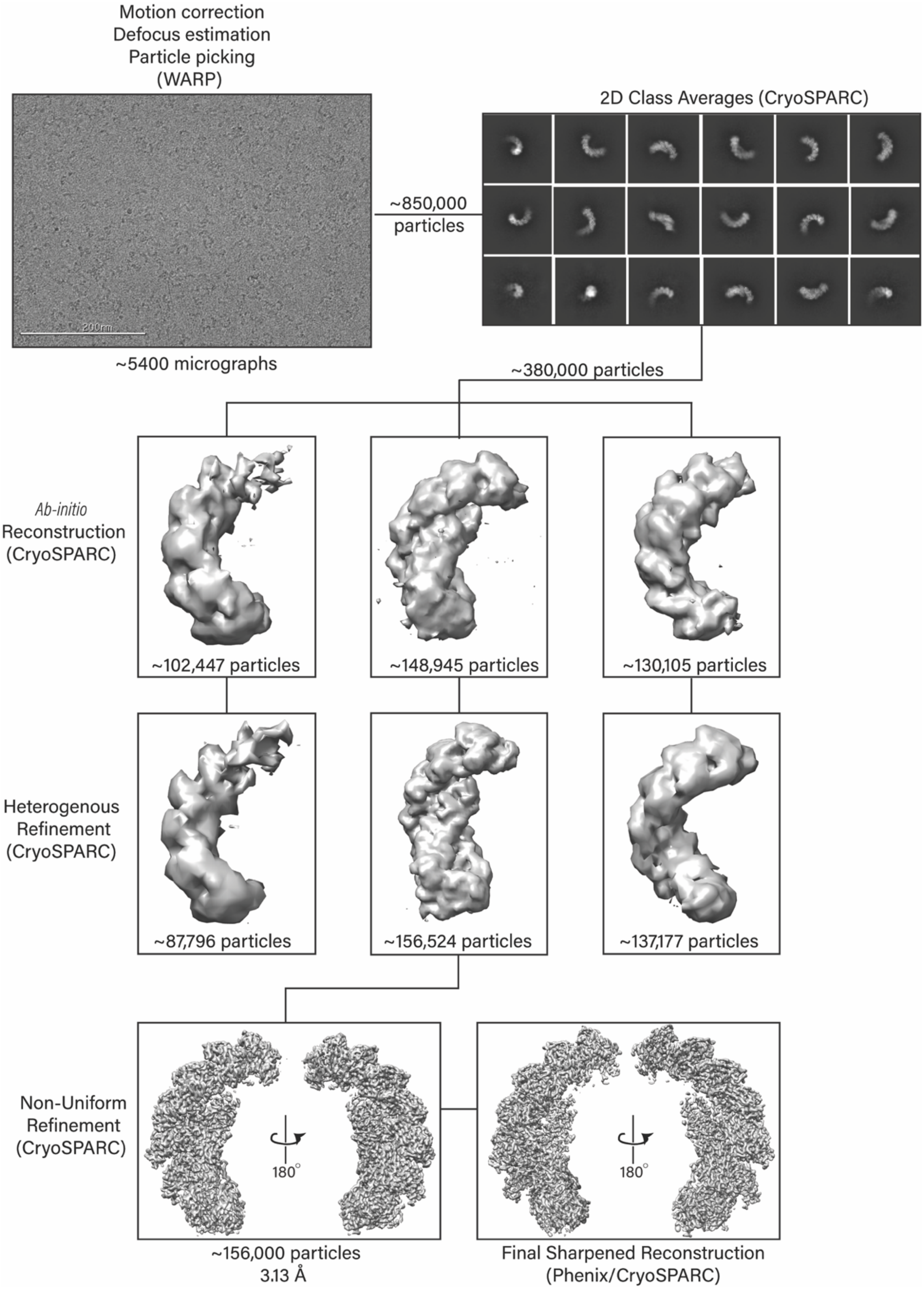

Supplementary Figure 2. Cryo-EM data processing workflow. This experiment was repeated once.

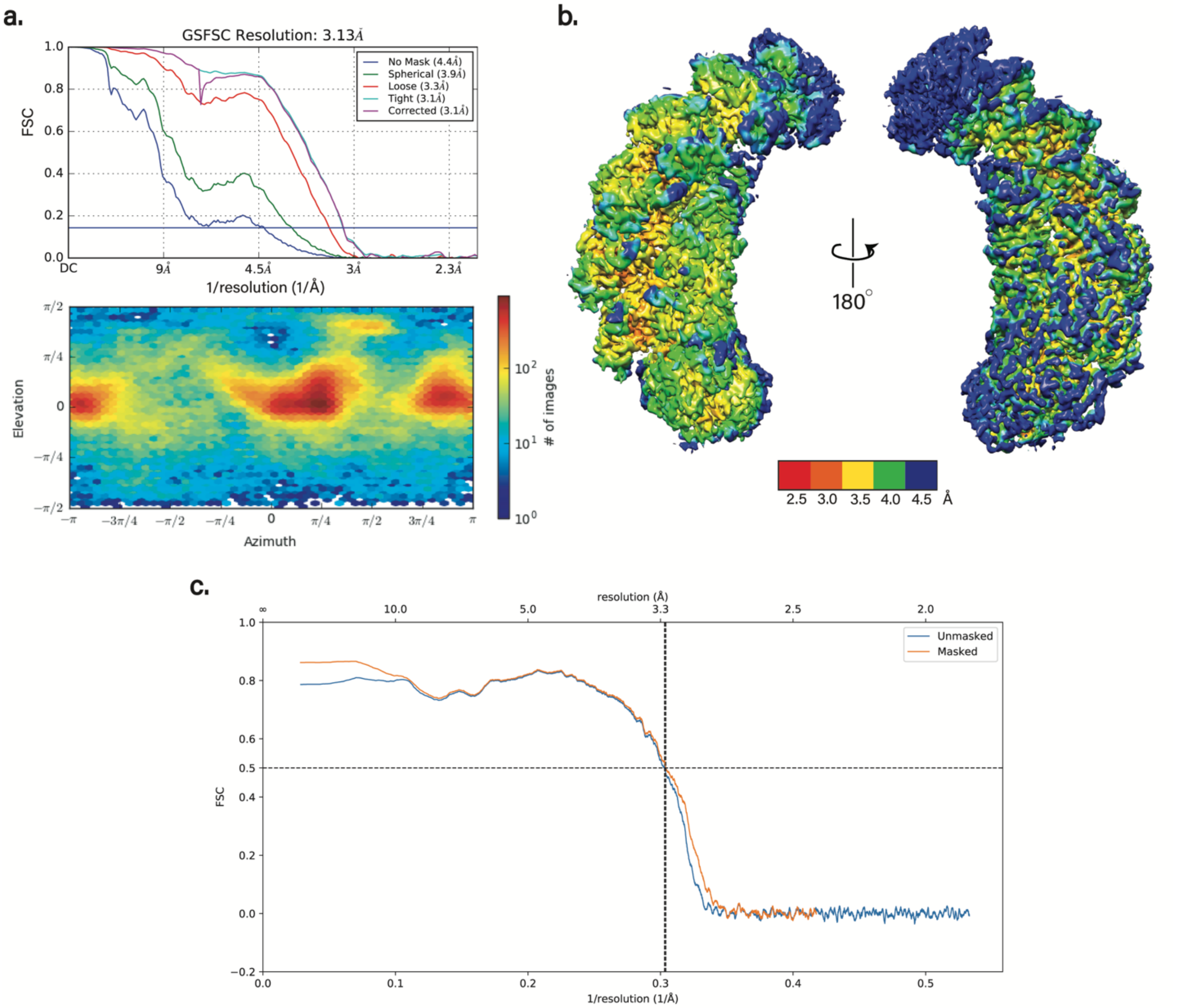

Supplementary Figure 3. Cryo-EM structure validation. a, FSC curves (top) and viewing direction distribution plot (bottom) for the type I-C Cascade. **b,** 3.13-Å resolution cryo-EM density of the type I-C Cascade colored by local resolution. **c,** Model-to-map FSC.

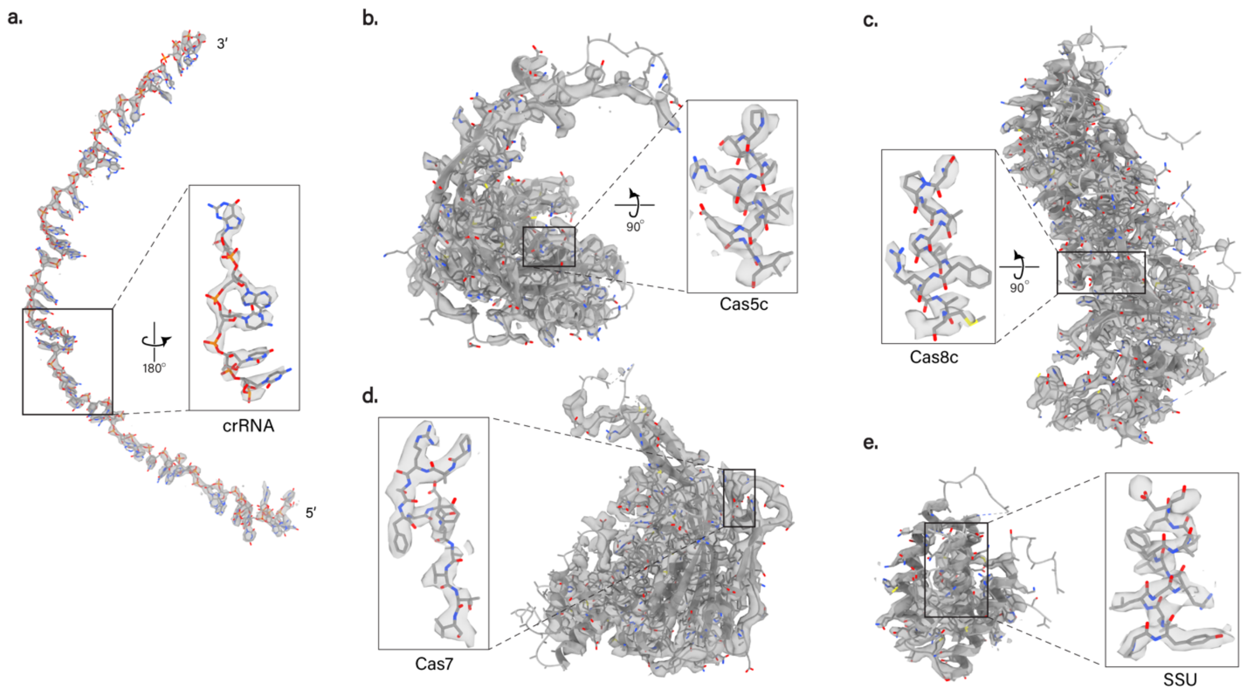

Supplementary Figure 4. EM density and corresponding model from crRNA (a), Cas5c (b), Cas8c (c), Cas7 (d), and SSU (e).

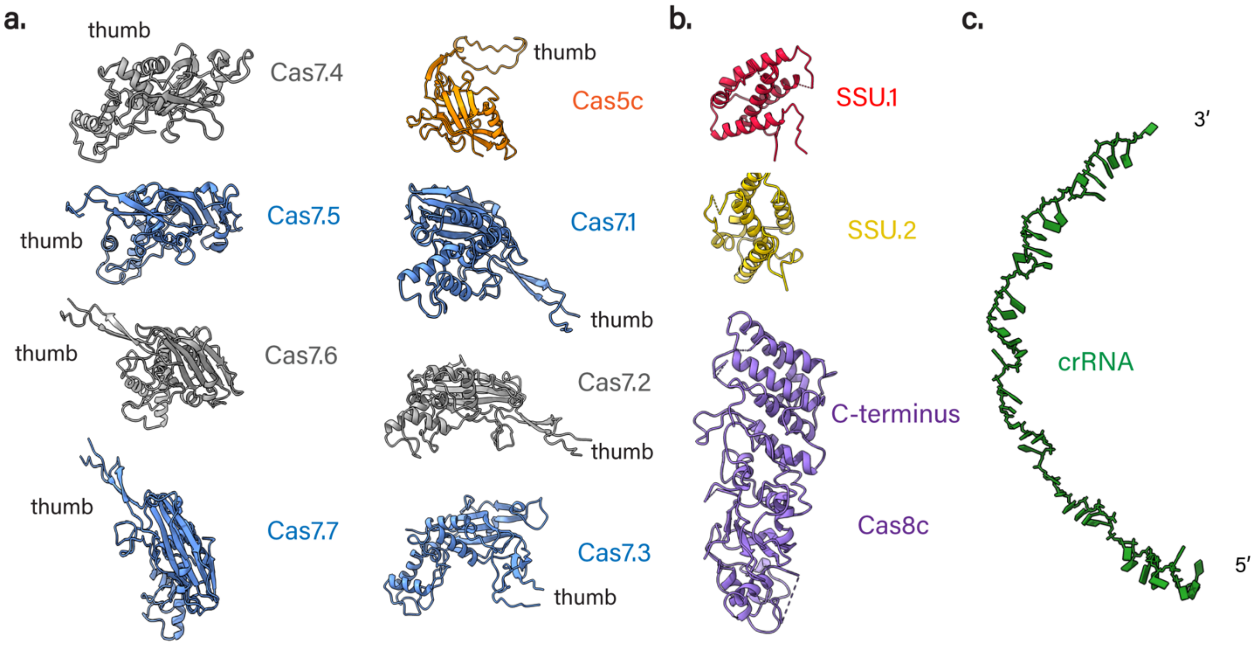

Supplementary Figure 5. The type I-C Cascade has a unique stoichiometry of subunits. a, Cas7 and Cas5c subunits have an extended beta-hairpin thumb and directly contact the crRNA. **b,** Two small subunits (SSU) sit in the belly of the complex and are produced from internal translation of the Cas8c c-terminus domain. **c,** All of the subunits assemble around the elongated crRNA.

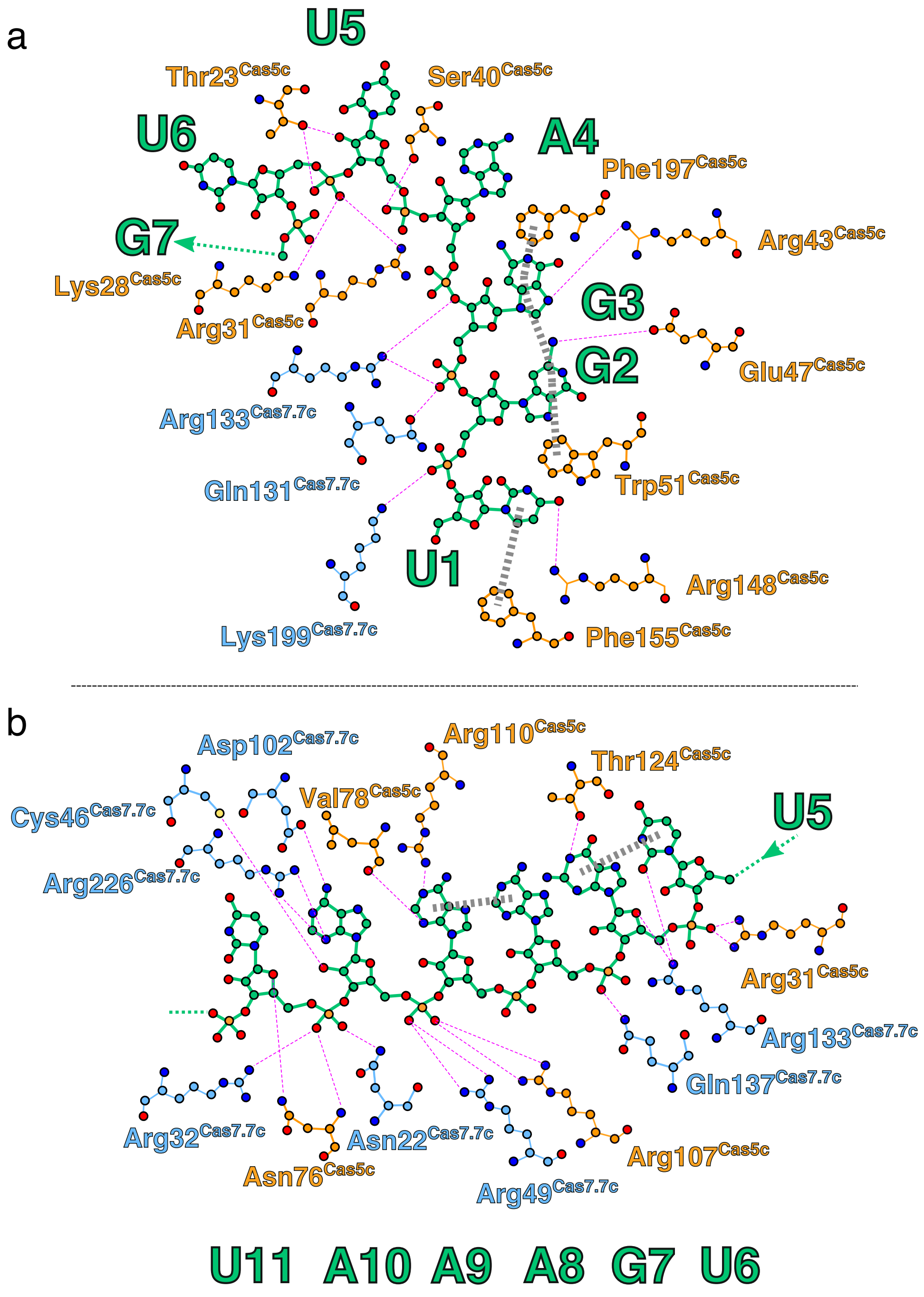

Supplementary Figure 6. Cas5c and Cas7.7 clamp around the crRNA 5'-handle. a, S40, T23, K28, and R31of Cas5c 'pinch' the phosphate groups within the crRNA backbone on either side of the U5 nucleobase. Cas5c residues W51 and F197 form a vice around crRNA nucleobases G2 and G3 through π-π stacking interactions, while U1 stacks against F155 and is further reinforced through non-specific interactions between Lys199, Arg133, and Gln131 of Cas7.7 (to G2, G3 and A4, respectively). **b,** Downstream of the U5 kink, U6-G7 and A8-A9 stack in doublets, providing rigidity and allowing the backbone to pivot and relieve the strained crRNA backbone conformation.

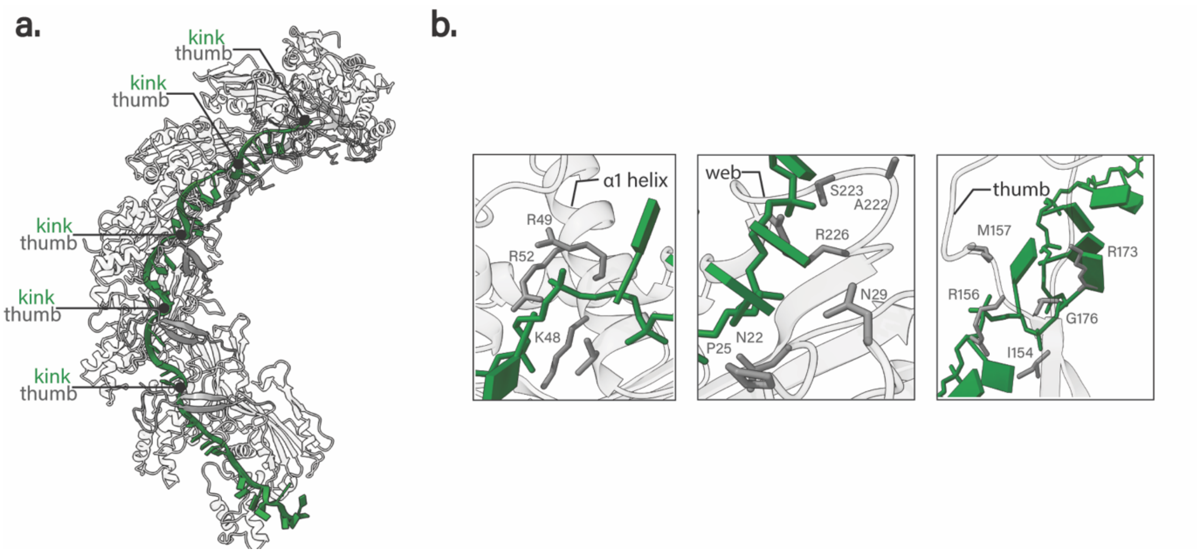

Supplementary Figure 7. The Type I-C Cas7 subunits introduce kinks in crRNA through non-specific interactions. **a,** Cas7 backbone subunits (grey) introduce kink in crRNA (green) through the extended beta-hairpin thumb that interacts with the adjacent Cas7 finger domain. **b,** Non-specific contacts (dark grey) with the crRNA phosphate backbone occur through the α1 helix (left) and web region (middle) located within the RRM domain and the thumb (right).

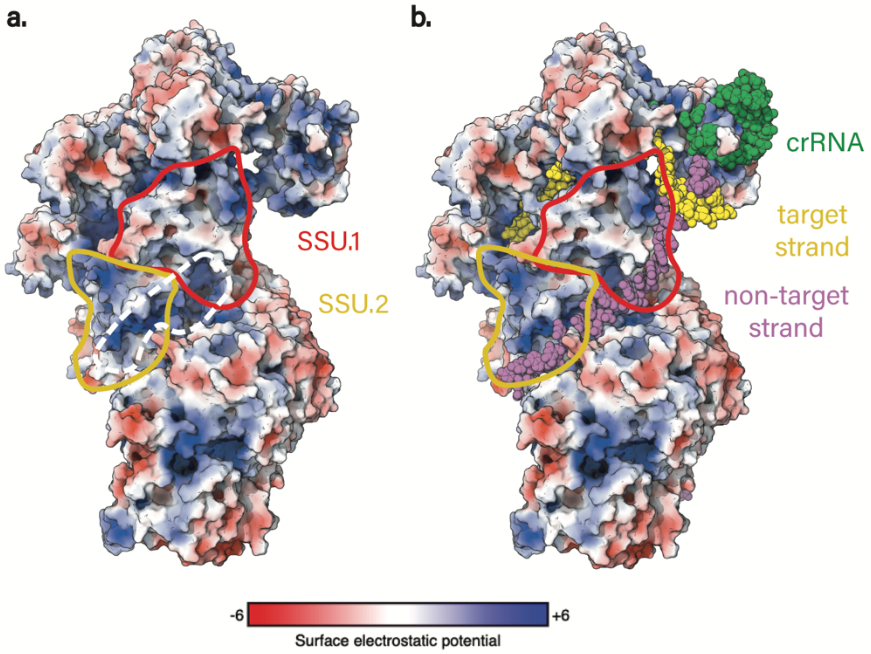

Supplementary Figure 8. Non-target strands are supported during R-loop formation through a positive channel. a, Similar to type I-C, the type I-E Cascade displays a positive channel (outlined in white) that runs along the length of the small subunits, SSU. **b,** The positive residues demonstrated in A support the non-target strand during R-loop formation.

Supplementary Table 1. Overview of type I-C Cascade and its subcomplexes identified by native mass spectrometry with in-source trapping.

Complex	Theoretical mass (Da)	Experimental mass (Da)	Deviation from expected mass
$Cas7_7Cas8c_1Cas5c_1SSU_2/crRNA_1$	368,546.4	371,960	0.93%
$Cas7_7Cas5c_1/crRNA_1$	272,580.4	275,230	0.97%
$Cas7_7/crRNA_1$	246,658.1	247,120	0.19%
$Cas7_6Cas5c_1/crRNA_1$	240,419.1	240,940	0.22%
$Cas8c_1$	67,989.7	69,030	1.53%
$Cas7_2$	64,322.6	64,37	0.07%
$Cas7_1$	32,161.3	32,180	0.06%
$Cas5c_1$	25,922.3	27,790	7.20%
SSU_1	13,988.2	13,900	0.63%
$crRNA_1$	21,528.9		

Supplementary Table 2. Cryo-EM data collection and refinement statistics

**EM data collection and reconstruction statistics**	
Protein	*D. vulgaris* Type I-C Cascade
EMBD	EMD-22876
Microscope	FEI Titan Krios
Voltage (kV)	300
Detector	Gatan K3
Magnification (nominal)	22500
Pixel size (Å/pix)	1.047
Flux (e^-/pix/sec)	8.0
Frames per exposure	30
Spherical aberration (mm)	2.7
Electron exposure (e^-/Å^2)	33
Defocus range (μm)	0.7-3.2
Micrographs collected	5399
Particles extracted/final	847,259/156,524
**Model refinement and validation statistics**	
PDB	7KHA
Symmetry imposed	C1
FSC threshold	0.5
Resolution threshold 0.143 FSC (Å) (masked/unmasked)	3.1/4.4
Composition<br>Amino Acids<br>Nucleotides	 2848<br>45
RMSD bond Lengths (Å)	0.009
RMSD angels (°)	0.950
Mean B-factors<br>Protein<br>Nucleotides	 62.80<br>48.57
Ramachandran<br>Favored (%)<br>Allowed (%)<br>Outliers (%)	 93.73<br>6.23<br>0.04
Rotamer outliers (%)	0.33
Clash score	16.59
C-beta outliers	.04
CaBLAM outliers	3.69
CC (mask)	0.75
MolProbity score	2.14
EMRinger	2.99
